# Prediction of growth parameters in the medicinal plant *Astragalus membranaceus* var. *mongholicus* (Bunge) P.K.Hsiao using Unmanned Aerial Vehicle multispectral measurement

**DOI:** 10.3389/fpls.2026.1931838

**Published:** 2026-09-07

**Authors:** Feng Pang, Haoyan Jiang, Tao Li, Haibin Guan, Yukai Zhao, Chuanling Zhang, Xin Jia, Yan Xue, Fengyu Han, Xiaoqin Wang, Yang Cao

**Affiliations:** 1 College of Pharmacy, Inner Mongolia Medical University, Hohhot, China; 2 Chifeng Academy of Agricultural and Animal Husbandry Sciences, Chifeng, China; 3 Inner Mongolia Tianqi Han and Mongolia Pharmaceutical Co. Ltd., Chifeng, China

**Keywords:** *A. membranaceus*, aboveground biomass, leaf area index, machine learning, underground biomass, Unmanned Aerial Vehicle multispectral remote sensing

## Abstract

Medicinal plants are critical sources of bioactive materials, yet efficient field-scale monitoring of their growth responses to water and nitrogen management remains limited. The present work evaluated Unmanned Aerial Vehicle (UAV) multispectral sensing for estimating leaf area index (LAI), underground fresh weight (UFW), aboveground fresh weight (AFW), underground dry weight (UDW), and aboveground dry weight (ADW) in *Astragalus membranaceus* var. *mongholicus* (Bunge) P.K.Hsiao (*A. membranaceus*) under 15 irrigation-nitrogen treatments spanning irrigation inputs of 15.0-30.0 mm per event and nitrogen application rates of 0–450 kg N ha⁻¹. UAV imagery and ground measurements were acquired from 45 plots at 60, 75, 90, 105, 120, and 135 days after sowing (DAS). Thirty-seven vegetation indices (VIs) were screened, and random forest (RF), support vector machine (SVM), back-propagation neural network (BP), genetic algorithm-optimized BP neural network (GA-BP), and decision-level fusion (DLF) models were evaluated with repeated random partitions. Growth responses were trait-dependent. W1N5 reached the peak final LAI (1.96), whereas W2N2 achieved the maximum UFW, AFW, UDW, and ADW (4363.94, 7406.00, 1354.50, and 2596.51 kg ha⁻¹, respectively). VI-trait associations changed with growth stage. GNDVI correlated most closely with LAI at 75 DAS (r = 0.78), and biomass-model performance was stronger during 90–120 DAS than at 60 DAS. DLF was retained in 24 of the 30 stage-specific models. In whole-season modeling, LAI was best estimated by BNDVI input with BP (R^2^ = 0.96; RMSE = 0.01), whereas UDW was best estimated by DVI input with DLF (R^2^ = 0.93; RMSE = 0.39). Stage-adaptive feature selection increased R^2^ by up to 38.60%. Overall, the results indicate that UAV multispectral sensing, stage-adaptive VI screening, and ensemble learning provide a practical framework for monitoring *A. membranaceus* growth and supporting precision irrigation-nitrogen management in medicinal plant production.

## 1 Introduction

*Astragalus membranaceus* var. *mongholicus* (Bunge) P.K.Hsiao (*A. membranaceus*) is a perennial medicinal herb in the genus *Astragalus* (Fabaceae). Its roots have been used in traditional Chinese medicine for more than 2,000 years and contain astragaloside IV, flavonoids, and other constituents associated with immunomodulatory, antioxidant, and metabolic-regulatory activity (Fu et al., 2014; Liu et al., 2022). In China, the annual cultivation area of *A. membranaceus* exceeds 2.27 × 10^4^ ha, with an estimated production of 8.25 × 10^7^ kg. Nevertheless, large-scale production remains constrained by inefficient water and fertilizer management, which can reduce yield and medicinal quality while increasing environmental pressure (Chen et al., 2016). Because medicinal plants are cultivated for defined therapeutic uses, field management must satisfy strict growth and quality requirements. Rising labor costs under rapid urbanization and an aging rural workforce accordingly increase the need for reliable precision-monitoring technologies (Li et al., 2022a, Li et al., 2022b).

Growth status is closely linked to yield formation, quality development, and resource-use efficiency in medicinal plant production. Timely growth information is accordingly critical for field management and for optimizing irrigation and nitrogen inputs (Li et al., 2025a). Leaf area index (LAI), defined as the ratio of total one-sided leaf area to unit ground surface area, is a standard indicator of leaf-area development, crop vigor, biomass, and yield potential. Fresh and dry weights of aboveground and underground organs provide direct evidence of biomass accumulation and root-shoot allocation. Conventional biomass assessment, however, still depends largely on destructive sampling. These measurements are accurate but labor-intensive, time-consuming, and unsuitable for repeated monitoring across large fields or multiple growth stages. Near-ground LAI instruments such as the LAI-2000, LAI-2200C, and TOP-1200 improve LAI measurement, yet they remain plot-scale, operator-sensitive, and inefficient for dynamic field monitoring (Yan et al., 2019). Correspondingly, Unmanned Aerial Vehicle (UAV) remote sensing offers a rapid, repeatable, and non-destructive route for acquiring plot-level multispectral reflectance at high spatial resolution (Maes and Steppe, 2019). These advantages explain why UAV platforms have become a core tool for high-throughput field phenotyping and precision-agriculture monitoring (Yang et al., 2017).

Multispectral imagery is commonly transformed into vegetation indices (VIs) before modeling. These indices condense band reflectance into variables associated with LAI, pigment status, water response, soil background, and biomass formation. In medicinal plants, UAV-derived VIs have mainly been used to diagnose nutritional status and monitor growth under cultivation treatments. Li et al. (2023) diagnosed nutrient deficiencies in *Ligusticum chuanxiong* using UAV multispectral time-series imagery and multiple classifiers, with the best model identifying N-deficient plants with 100% accuracy and P/K-deficient plants with 92.4% accuracy. Zhang et al. (2025) integrated UAV-derived VIs and phenotypic indicators to monitor *Glycyrrhiza uralensis* under irrigation-nitrogen treatments, reaching R^2^ values of 0.94 for phenotypic indicators and 0.87 for VI-based yield prediction. The application scope has also moved toward harvest-related targets. In *L. chuanxiong*, UAV multispectral features were used to estimate rhizome fresh and dry weights and to classify ferulic-acid-based quality; in industrial poppy, aerial spectral information was used to predict thebaine alkaloid concentration (Li et al., 2025b; Iqbal et al., 2020). Accordingly, medicinal-crop UAV studies now cover nutritional diagnosis, growth monitoring, and harvest-related quality assessment, whereas continuous inversion of multiple growth traits under water-nitrogen regulation remains insufficiently addressed. In organic oil rose, UAV-derived VIs and soil attributes supported early yield prediction, with the best machine-learning models attaining accuracies of 88.8%-93.1% (Demir et al., 2024).

Beyond plot-scale crop traits, multispectral and geospatial workflows have been applied to agricultural land classification, land-suitability assessment, and soil organic-carbon mapping (Demir, 2024a, 2024b; Demir and Çağ, 2025). Related satellite studies have mapped burned vegetation and post-fire erosion dynamics (Demir, 2023; Demir and Başayiğit, 2024; Demir and Dursun, 2024; Dursun et al., 2025). Although these landscape applications address different targets and spatial scales, they demonstrate that spectral interpretation depends on land cover, soil properties, and environmental context. This broader evidence reinforces the need for crop- and stage-specific calibration when UAV reflectance is used for quantitative growth monitoring.

Extending these applications to root biomass estimation depends on a biologically meaningful relationship between aboveground traits and belowground biomass accumulation. This relationship can be interpreted within a whole-plant source-sink framework. LAI represents the leaf surface available for light interception, whereas pigment-sensitive reflectance provides information on leaf physiological status (Gitelson et al., 2003). Photoassimilates produced by mature leaves are exported through the phloem to sink organs, including roots, and their allocation among organs changes with plant development and resource availability (Ainsworth and Bush, 2011; Poorter et al., 2012). Consequently, underground dry weight (UDW) primarily reflects cumulative dry matter deposition in roots, whereas underground fresh weight (UFW) integrates dry matter accumulation with tissue hydration at sampling. Resource availability can therefore affect above- and belowground growth simultaneously. In *A. membranaceus* var. *mongholicus*, nitrogen fertilization altered plant height, root morphology, aboveground biomass, and root biomass (Wang et al., 2020). Explainable UAV modeling across maize, millet, and sorghum further identified leaf area as the dominant aboveground predictor of root biomass, while leaf water content, chlorophyll-related traits, and LAI provided additional crop-specific information (Shan et al., 2026). LAI and multispectral VIs may therefore provide indirect information on UFW and UDW, although these associations may vary across growth stages and irrigation-nitrogen treatments as biomass allocation and tissue hydration change during development.

LAI-focused work points first to the role of feature construction (Chen et al., 2025). Hussain et al. (2025) compared UAV-derived VIs and machine-learning models in sweet corn and green beans, and RF and extreme gradient boosting achieved R^2^ values of 0.90 for sweet corn. Other LAI studies using spectral-textural inputs likewise report that feature construction affects estimation accuracy (Sun et al., 2023; Fan et al., 2024; Zou et al., 2024). Biomass and yield estimation provide a second line of evidence (Li et al., 2020). For biomass estimation, Dai et al. (2024) combined VIs, plant height, coverage metrics, and temporal information across rice phenological stages. Related biomass and yield studies likewise show that temporal information, feature fusion, and algorithm selection affect UAV-based trait inversion (Amorim et al., 2022; Maimaitijiang et al., 2020). Together, these studies show that feature construction and algorithm selection should be evaluated across traits and growth stages rather than treated as fixed choices.

Machine-learning algorithms are well suited to UAV spectral-trait modeling because they can capture nonlinear responses that simple empirical regressions often miss. Zheng et al. (2022) extracted 25 spectral variables from UAV multispectral imagery for strawberry dry biomass prediction and compared six regression methods. The artificial neural network yielded the best accuracy (R^2^ = 0.89-0.93; RMSE = 7.16-8.98 g), and adding VIs increased the R^2^ of other models from 0.77-0.80 to 0.83-0.86. Shi et al. (2025) developed a random forest model with organ-specific feature selection for rice aboveground biomass estimation, achieving R^2^ = 0.85 and RMSE = 196.55 g m⁻² in the validation set. This comparison suggests that individual learners can perform well, although their accuracy remains shaped by feature construction, target traits, and sample structure. Ensemble strategies have accordingly been introduced to reduce reliance on a single learner. Cheng et al. (2024) reconstructed spectral indices from UAV multispectral data and integrated seven machine-learning models with Bayesian model averaging, obtaining R^2^ = 0.78-0.84 and RMSE = 810–890 kg ha⁻¹ for aboveground biomass in film-mulched wheat and maize. Compared with individual learners, integrated modeling can better exploit complementary algorithm behavior. Nevertheless, medicinal-plant studies that simultaneously address multiple growth traits, stage-adaptive VI selection, multi-algorithm comparison, and fusion-based modeling remain scarce.

Acquisition timing adds another layer of uncertainty to UAV-based trait estimation. Prey et al. (2022) reported that the milk-ripeness stage was generally the most reliable single stage for winter wheat yield prediction, with maximum R^2^ values of 0.81 and 0.68 in two drought-affected trials; multi-date combinations further improved prediction. For dynamic LAI estimation, Qiao et al. (2022) demonstrated that fusing UAV-derived morphological parameters with VIs improved maize LAI estimation across six growth stages, with an overall R^2^ of 0.943. In rice aboveground biomass estimation, Dai et al. (2024) found that UAV-derived feature correlations changed across phenological stages, and the optimal plot-scale model reached R^2^ = 0.88, RMSE = 1111–1131 kg ha⁻¹, and normalized RMSE (NRMSE) = 9.76%-9.94%. Chatterjee et al. (2025) further reported that VI-based LAI models showed inconsistency and saturation at later stages, whereas reflectance-based models outperformed VI-based models by 5%-15% at mid- to late-vegetative stages and by 25% at silking. Thus, growth stage should be treated as a factor controlling spectral sensitivity, feature stability, and model transferability rather than merely as a sampling label. Such timing effects are particularly relevant for medicinal-plant monitoring, where LAI and multiple biomass traits must be estimated together rather than treated as a single spectral target.

Accordingly, the present work collected UAV multispectral observations and ground measurements at six growth stages (60, 75, 90, 105, 120, and 135 days after sowing [DAS]) under 15 irrigation-nitrogen treatments. The treatments were formed by combining three irrigation amounts per event (W1, 30.0 mm; W2, 22.5 mm; and W3, 15.0 mm) with five nitrogen application rates (N1-N5: 0, 150, 225, 300, and 450 kg N ha⁻¹). LAI, UFW, AFW, UDW, and ADW were used as target traits. Thirty-seven vegetation indices (VIs) were screened by trait-stage correlations, and random forest (RF), support vector machine (SVM), back-propagation neural network (BP), genetic algorithm-optimized back-propagation neural network (GA-BP), and decision-level fusion (DLF) were compared under stage-specific and whole-season frameworks. The objectives were to: (1) characterize treatment-driven growth variation; (2) identify trait- and stage-specific VI responses; (3) evaluate retained VI-model combinations and fusion performance; (4) provide a remote-sensing reference for precision irrigation-nitrogen management in *A. membranaceus* production.

## 2 Materials and methods

### 2.1 Experimental site and field design

**Figure 1:**
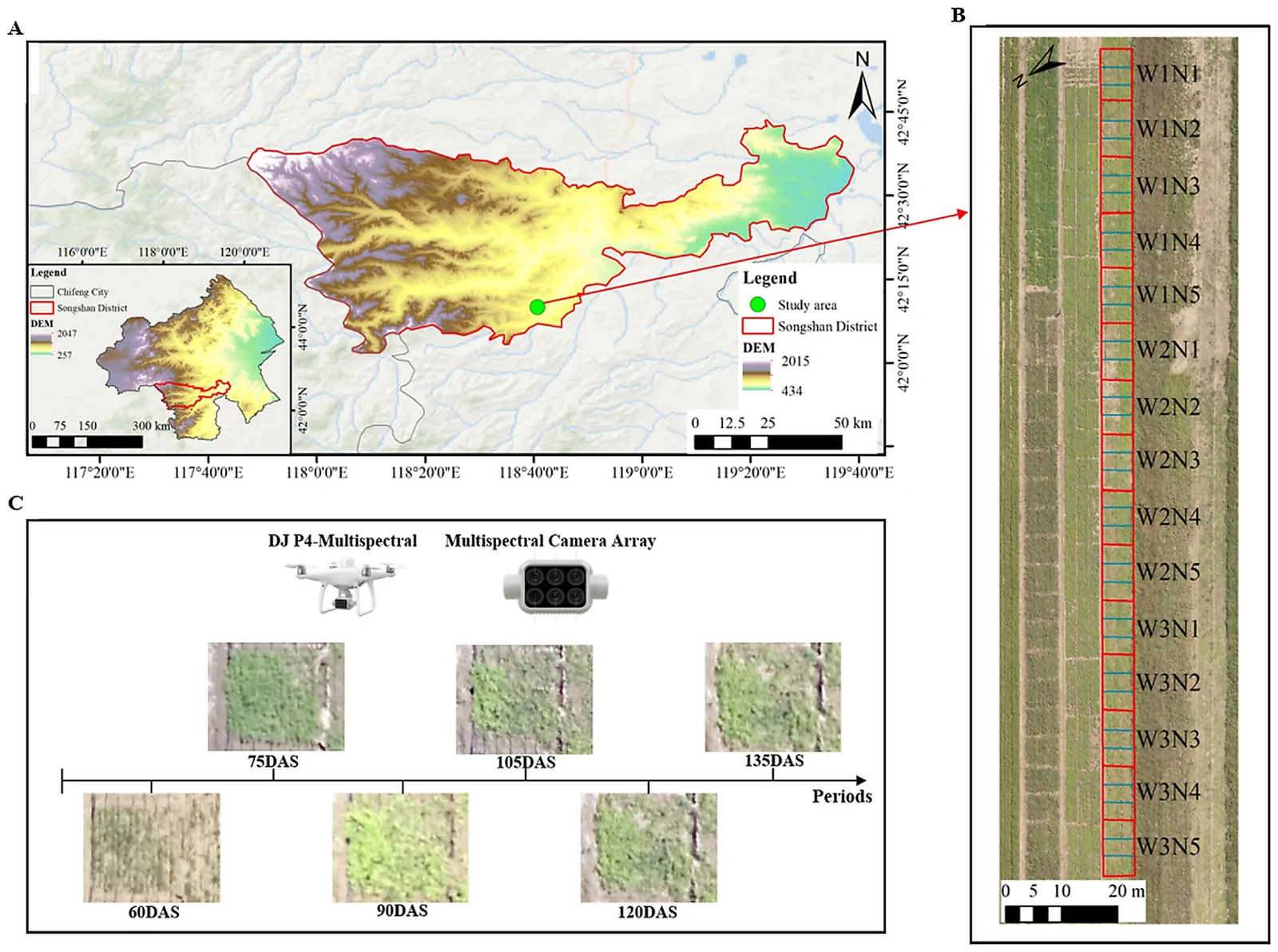
Location of the study area and orthophoto of the field experimental site. **(A)** Geographical location of the study area; **(B)** Experimental plots with three irrigation levels and five nitrogen levels during the 2024 growing season; **(C)** Field acquisition of UAV multispectral images.

The field experiment was conducted in 2024 in Chengzi Township, Songshan District, Chifeng City, Inner Mongolia, China (42.16°N, 118.72°E) (**Figure 1**). The region has a mid-temperate continental monsoon climate, with a mean annual temperature of 6.2 °C, mean annual precipitation of approximately 335 mm, and a frost-free period of about 130 d. The experimental field was flat and equipped with a drip irrigation system. The soil type was cinnamon soil, with an organic matter content of 1.5%-2.0%, which is suitable for *A. membranaceus* cultivation.

A two-factor orthogonal field plot experiment was established with irrigation and nitrogen application as the experimental factors. Treatment ranges were selected with reference to the technical code for *A. membranaceus* var. *mongholicus* cultivation in eastern Inner Mongolia (Inner Mongolia Administration for Market Regulation, 2021), which recommends 225–300 kg N ha⁻¹, and local production practice. The N treatments bracketed this recommendation, comprising zero input (N1), a lower rate (N2), rates within the recommended range (N3 and N4), and a higher rate (N5). The irrigation treatments represented 100%, 75%, and 50% of a 30-mm reference event: W1 = 30 mm (high), W2 = 22.5 mm (moderate), and W3 = 15 mm (low), equivalent to 270, 202.5, and 135 L per 9 m² plot, respectively. Phosphorus and potassium fertilizers were applied uniformly before sowing at 180 kg ha⁻¹ P_2_O_5_ and 75 kg ha⁻¹ K_2_O, respectively. Nitrogen was supplied at five rates: N1 = 0 kg ha⁻¹ N, N2 = 150 kg ha⁻¹ N, N3 = 225 kg ha⁻¹ N, N4 = 300 kg ha⁻¹ N, and N5 = 450 kg ha⁻¹ N. The design comprised 15 irrigation-nitrogen combinations with three replicates, giving 45 plots.

Each plot covered 9 m² and was separated from adjacent plots by a 1 m interval. Drainage ditches and guard rows reduced lateral water movement and edge effects. Seeds of *A. membranaceus* were sown on 20 May 2024, defined as 0 DAS, at a density of 35 plants m⁻² and a row spacing of 0.25 m. The drip-irrigation system used 16-mm-diameter laterals, with emitters spaced 0.30 m apart and a nominal discharge of 2.0 L h⁻¹. Operating pressure was maintained at 0.10-0.15 MPa. Treatment irrigation was applied approximately 5 d before each paired ground and UAV observation. The target water volume for each plot was delivered through the drip system and controlled using a flow meter and irrigation duration. Fertilizers were applied once as basal fertilizer before sowing, and all non-treatment management followed local cultivation practice.

Ground measurements and UAV image acquisition were conducted on the same dates: 60 DAS (19 July), 75 DAS (3 August), 90 DAS (18 August), 105 DAS (2 September), 120 DAS (17 September), and 135 DAS (2 October).

### 2.2 Ground measurements and UAV image acquisition

#### 2.2.1 Measurements of LAI and biomass traits

LAI was measured in each plot using a TOP-1200 plant canopy analyzer (Top Cloud Agricultural Technology Co., Ltd., Hangzhou, China). At each observation date, five independent LAI readings were recorded from representative central positions within each plot, with the sensor probe kept perpendicular to the canopy surface at approximately 1.2 m above the canopy top; the arithmetic mean was used as plot-level LAI. For biomass determination, five plants were sampled from the central area of each plot while avoiding border rows and previously sampled positions. For each plant, a 60-cm-diameter excavation area (30-cm radius) centered on the root crown was opened to a depth of 60 cm. Soil was loosened progressively from the perimeter toward the plant, and the main root and attached lateral roots within the excavation volume were recovered as intact as possible to minimize biomass loss from root breakage. Plants were then separated into aboveground and underground organs at the root collar. Roots were gently washed with clean water to remove adhering soil, and detached visible root fragments were retained with the corresponding sample. After surface moisture was removed, fresh aboveground and underground weights were measured with an electronic balance (0.01 g) and recorded as AFW and UFW, respectively, in g plant⁻¹. Samples were then oven-dried at 65 °C to constant weight, and dry aboveground and underground weights were recorded as ADW and UDW, respectively. Plot-level biomass values were calculated as the mean of the five sampled plants. For treatment comparison and figure preparation, biomass was converted to an area basis as follows:

(1) B=w×D×10

where B denotes UFW, AFW, UDW, or ADW on an area basis (kg ha⁻¹), w is the plot-level mean individual weight (g plant⁻¹), D is the planting density (35 plants m⁻²), and 10 converts g m⁻² to kg ha⁻¹.

#### 2.2.2 UAV multispectral image acquisition

UAV multispectral images were acquired with a DJI Phantom 4 Multispectral platform (DJI Technology Co., Ltd., Shenzhen, China). The imaging system includes one red-green-blue (RGB) camera and five monochrome multispectral cameras covering blue (B, 450 ± 16 nm), green (G, 560 ± 16 nm), red (R, 650 ± 16 nm), red-edge (RE, 730 ± 16 nm), and near-infrared (NIR, 840 ± 26 nm) bands. An integrated spectral sunlight sensor recorded incident irradiance for each exposure.

Flights were synchronized with ground sampling at 60, 75, 90, 105, 120, and 135 DAS. Missions were planned in DJI GS Pro and conducted between 11:00 and 13:00 under clear or near-clear conditions, with wind speed below Beaufort force 3. Flight altitude and speed were 50 m and 5 m s⁻¹, respectively, and forward and side overlaps were both 80%. Immediately before each mission, images of 25%, 50%, and 75% reflectance panels were acquired under the same illumination while avoiding shadows on the panels. The same flight parameters and acquisition window were maintained across all dates.

#### 2.2.3 Image processing and region of interest extraction

Raw multispectral images were recorded as digital numbers (DNs) and processed in Pix4Dmapper. Calibration-panel images were imported with each flight dataset, and a central homogeneous area of each panel was marked using the manufacturer-supplied reflectance factor for each band. During reflectance-map generation, the “Camera and Sun Irradiance” radiometric-correction option was applied. Camera calibration metadata and irradiance measurements recorded by the spectral sunlight sensor were used to correct sensor effects and normalize illumination variation among images. Georeferenced orthomosaics and reflectance maps were then generated for the five spectral bands. For each plot, a central region of interest (ROI) was manually delineated according to plot boundaries. Borders, drainage ditches, visible bare soil, shadows, weeds, and abnormal pixels were excluded, and mean B, G, R, RE, and NIR reflectance values were extracted from the retained ROI.

Vegetation indices (VIs) listed in **Table 1** were calculated from plot-level mean reflectance for each plot and observation date.

**Table 1:** Vegetation indices used in this study.

VIs	Formula	Reference
BRVI	R/B	Pádua et al., 2019
GRRI	R/G	Xie et al., 2024
NDI	(R − G)/(R + G + 0.01)	El-Hendawy et al., 2019
NDWI	(G − NIR)/(G + NIR)	Wu et al., 2009
GBNDVI	(G − B)/(G + B)	Han et al., 2022
LCI	(NIR − RE)/(NIR + R)	Hunt et al., 2011
WDRVI2	(0.10 × NIR − R)/(0.10 × NIR + R)	Cao et al., 2020
NDRE	(NIR − RE)/(NIR + RE)	Shu et al., 2021
WDRVI1	(0.05 × NIR − R)/(0.05 × NIR + R)	Cao et al., 2020
EVI	2.5 × (NIR − R)/(NIR + 6R − 7.5B + 1)	Vescovo et al., 2012
MSR	[(NIR/R) − 1]/√[(NIR/R) + 1]	Vescovo et al., 2012
NLI	(NIR × NIR − R)/(NIR × NIR + R)	Zhang et al., 2021
EXG	2G − B − R	Xie et al., 2024
EMSR	[(NIR/RE) − 1]/√[(NIR/RE) + 1]	Cao et al., 2013
DVI	NIR − R	Gao et al., 2016
SAVI	1.5 × (NIR − R)/(NIR + R + 0.5)	Argolo dos Santos et al., 2020
OSAVI	1.16 × (NIR − R)/(NIR + R + 0.16)	Rondeaux et al., 1996
RVI	NIR/R	Gao et al., 2016
RERDVI	(NIR − RE)/√(NIR + RE)	Cao et al., 2013
RDVI	(NIR − R)/√(NIR + R)	Vescovo et al., 2012
ENDVI	[(NIR + G) − 2B]/[(NIR + G) + 2B]	Strong et al., 2017
ESAVI	[(NIR − R)/(NIR + R + L)] × (1 + L)	Qi et al., 1994
BNDVI	(NIR − B)/(NIR + B)	Samsuddin Sah et al., 2023
BVI	NIR/B	Matyukira and Mhangara, 2024
MCARI	[(RE − R) − 0.2(RE − G)] × (RE/R)	Tamilmounika et al., 2024
GRVI	NIR/G	Sapkota and Paudyal, 2023
ERVI	2.5 × (NIR − R)/(NIR + 6R − 7.5B + 1) × (NIR/R)	Zhou et al., 2023
EDVI	NIR − RE	Zhang et al., 2025
GNDVI	(NIR − G)/(NIR + G)	Theau et al., 2021
GLI	(2G − R − B)/(2G + R + B)	Kozub et al., 2017
GLA	(2G − R + B)/(2G + R + B)	Xie et al., 2024
NDVI	(NIR − R)/(NIR + R)	Gao et al., 2016
WDRVI3	(0.20 × NIR − R)/(0.20 × NIR + R)	Cao et al., 2020
WDRVI4	(0.50 × NIR − R)/(0.50 × NIR + R)	Cao et al., 2020
VARI	(G − R)/(G + R − B)	Lima et al., 2024
GI	(G − R)/(G + R)	Lima et al., 2024
NDGI	(G − NIR)/(NIR + G)	Markov et al., 2025

B, G, R, RE, and NIR denote blue, green, red, red-edge, and near-infrared reflectance, respectively.

### 2.3 Correlation analysis and candidate VI selection

Pearson correlation analysis evaluated relationships between each VI and the five measured traits (LAI, UFW, AFW, UDW, and ADW). Stage-specific correlations were calculated separately at 60, 75, 90, 105, 120, and 135 DAS using 45 plot-level observations per date. Whole-season correlations were calculated after pooling observations from all six dates (n = 270).

For each target trait, VIs were ranked by the absolute Pearson correlation coefficient. At both stage-specific and whole-season scales, the three highest-ranked VIs were screened as candidate inputs for model construction.

### 2.4 Single-VI model construction and evaluation

#### 2.4.1 Single-VI model construction

**Figure 2:**
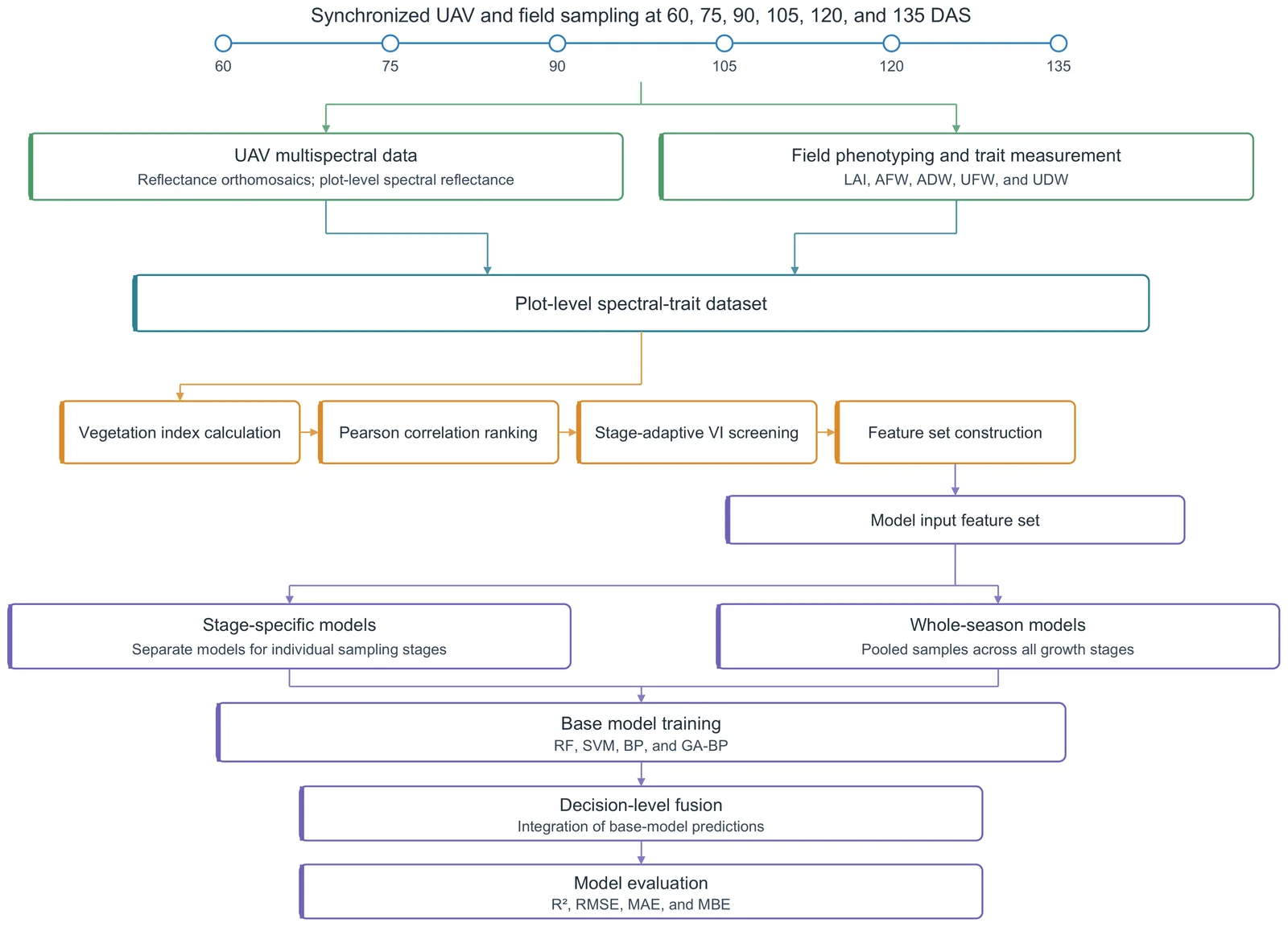
Workflow of UAV multispectral monitoring for *A. membranaceus* growth traits, including field sampling, UAV image acquisition, vegetation index calculation and screening, single-index model construction, multi-algorithm comparison, decision-level fusion modeling, and model evaluation.

Prediction models were constructed from the candidate VIs selected in Section 2.3. Each modeling case combined one target trait, one candidate VI, and one algorithm, with measured LAI, UFW, AFW, UDW, or ADW as the response variable. Retained models were selected by comparing validation performance under the same VI-screening rule, and the overall workflow is summarized in **Figure 2**.

To limit split-specific bias, each trait-VI-algorithm case was evaluated with 50 repeated random partitions. In each repetition, data were divided into training, validation, and test subsets at a 70:15:15 ratio. The training subset fitted the model, the validation subset tuned parameters, estimated fusion weights, and determined retained performance, and the test subset remained unused for model retention. Predictor and response variables were normalized to 0–1 using training-subset parameters to reduce scale effects and avoid information leakage; these parameters were then applied to the validation and test subsets:

(2) $$x_{i}^{*}=\frac{x_{i}-x_{\min,\text{ train}}}{x_{\max,\text{ train}}-x_{\min,\text{ train}}}$$

where x_i_ is the original value, x_i_* is the normalized value, and x_min_,train and x_max_,train are the minimum and maximum values of the corresponding variable in the training subset, respectively.

Five algorithms were evaluated: RF, SVM, BP, GA-BP, and DLF. RF and SVM represented tree- and kernel-based learners, whereas BP and GA-BP represented neural-network and genetic-optimization approaches. RF, SVM, BP, and GA-BP were first trained as individual learners, and DLF integrated their prediction outputs.

All models were developed in a consistent computing environment, and hyperparameters were tuned only on the validation subset. Based on the recorded modeling protocol, the final settings were as follows: RF used 200 decision trees; SVM used a radial basis function kernel with C = 4.0 and gamma = 0.8; BP used one hidden layer with five nodes; and GA-BP used the same BP architecture with 50 genetic-algorithm iterations. Stage-specific models used 31 training, 7 validation, and 7 test samples from each 45-plot date. Whole-season models used 189 training, 41 validation, and 40 test samples from the pooled 270 observations.

#### 2.4.2 Decision-level fusion

DLF integrated predictions from RF, SVM, BP, and GA-BP at the prediction-output level. For the i-th sample, the fused prediction was calculated as:

(3) $${\hat{y}}_{i,\text{DLF}}=\sum_{k=1}^{K}w_{k}{\hat{y}}_{ik}$$

where ŷi,DLF is the fused prediction, ŷik is the prediction from the k-th individual model for the i-th sample, wk is the corresponding weight, and K is the number of individual models in the fusion.

Model weights were derived from validation-set performance and normalized as follows:

(4) $$w_{k}=\frac{S_{k}}{\sum_{k=1}^{K}S_{k}}$$

where Sk is the validation-set performance score of the k-th individual model. Here, Sk was calculated from the non-negative validation-set coefficient of determination:

(5) $$S_{k}=\max(R_{val,k}^{2},0)+\epsilon$$

where R^2^val,k is the validation-set coefficient of determination for the k-th individual model, and epsilon is a small positive constant used to avoid zero weights. This procedure assigns larger weights to better-performing individual models and yields one fused prediction for each sample.

#### 2.4.3 Model evaluation

Model performance was evaluated using the coefficient of determination (R^2^), root mean square error (RMSE), mean absolute error (MAE), and mean bias error (MBE). The formulas were as follows:

(6) $${\text{R}}^{2}=1-\frac{\sum_{\text{i}=1}^{\text{n}}{\left({\text{y}}_{\text{i}}-{\hat{\text{y}}}_{\text{i}}\right)}^{2}}{\sum_{\text{i}=1}^{\text{n}}{\left({\text{y}}_{\text{i}}-\bar{\text{y}}\right)}^{2}}$$

(7) $$\text{RMSE}=\sqrt{\frac{1}{n}\sum_{i=1}^{n}{\left(y_{i}-{\hat{y}}_{i}\right)}^{2}}$$

(8) $$\text{MAE}=\frac{1}{n}\sum_{i=1}^{n}\left|y_{i}-{\hat{y}}_{i}\right|$$

(9) $$\text{MBE}=\frac{1}{n}\sum_{i=1}^{n}\left({\hat{y}}_{i}-y_{i}\right)$$

In these equations, yi and ŷi denote the observed and predicted values for the i-th sample, respectively; $\hat{\text{y}}$ is the mean observed value; and n is the sample size. R^2^ describes goodness of fit, RMSE and MAE quantify prediction error, and MBE indicates prediction bias.

## 3 Results

### 3.1 Temporal variation in LAI and biomass traits under irrigation-nitrogen treatments

**Figure 3:**
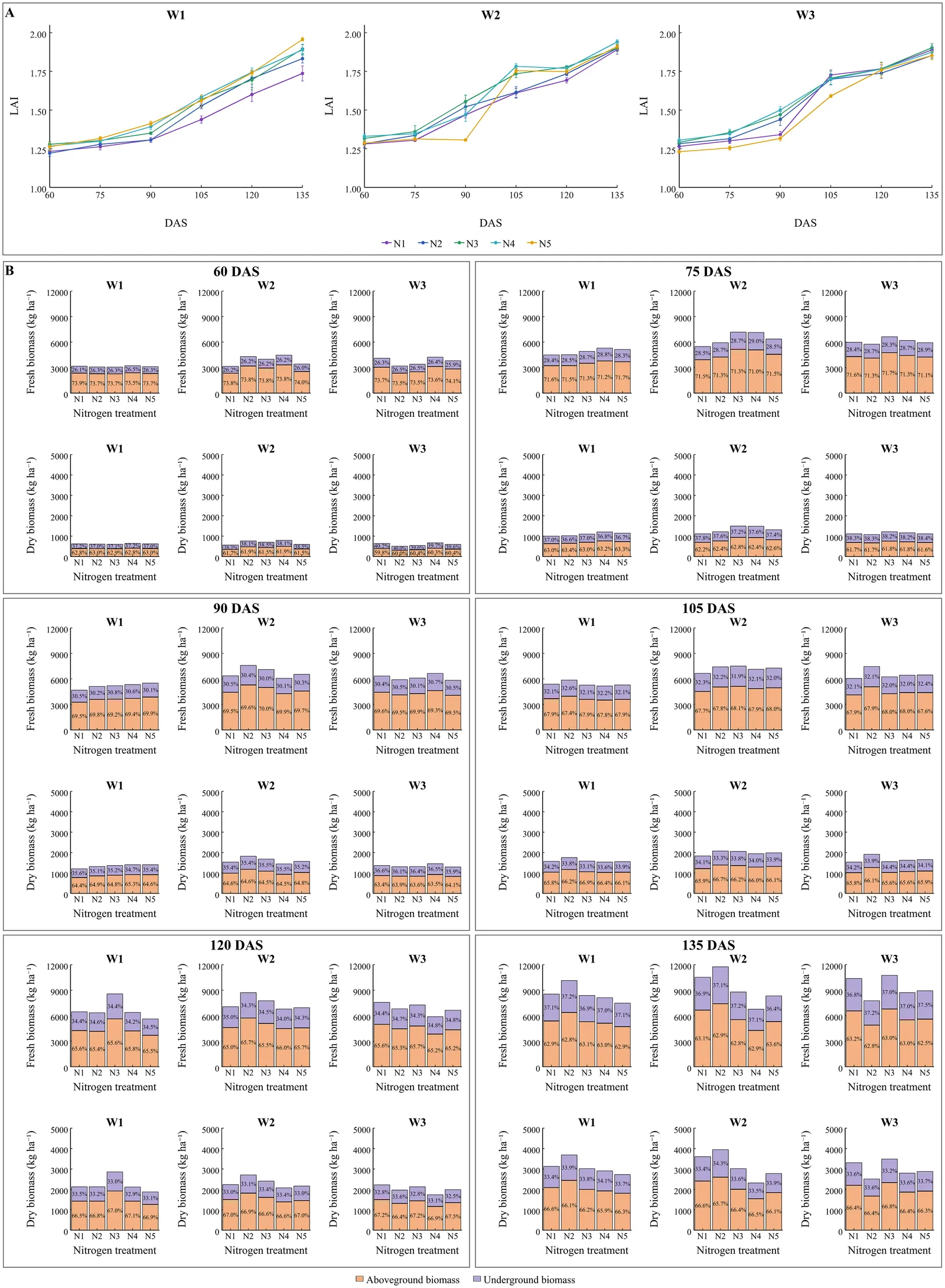
Temporal variation in LAI and biomass of *A. membranaceus* under different irrigation-nitrogen treatments across six growth stages. **(A)** Changes in LAI under the three irrigation levels (W1-W3). Colored lines and symbols denote the five nitrogen levels (N1-N5), and error bars represent the standard error of the mean. **(B)** Aboveground and underground fresh and dry biomass at 60, 75, 90, 105, 120, and 135 DAS. Within each growth stage, treatments are arranged according to irrigation level from W1 to W3. Orange and purple bar segments represent aboveground and underground biomass, respectively, and the values within each segment indicate its percentage contribution to total fresh or dry biomass.

LAI increased progressively across the six observation dates under all 15 irrigation-nitrogen treatments (**Figure 3A**). Differences among treatments were relatively small at 60 and 75 DAS but became increasingly pronounced after 90 DAS. At 135 DAS, LAI ranged from 1.74 to 1.96, with the highest value recorded under W1N5 and the lowest under W1N1.

Fresh and dry biomass generally increased with plant development, although the relative performance of the 15 irrigation-nitrogen treatments varied among growth stages (**Figure 3B**). Across all treatments and observation dates, aboveground tissues accounted for 62.55-74.13% of total fresh biomass and 59.83-67.54% of total dry biomass. At 135 DAS, W2N2 produced the highest UFW, AFW, UDW, and ADW values, reaching 4363.94, 7406.00, 1354.50, and 2596.51 kg ha⁻¹, respectively, whereas W2N4 exhibited the lowest final biomass values. Overall, the highest LAI was observed under W1N5, while the greatest fresh and dry biomass accumulation occurred under W2N2.

### 3.2 Correlations between vegetation indices and agronomic traits

**Figure 4:**
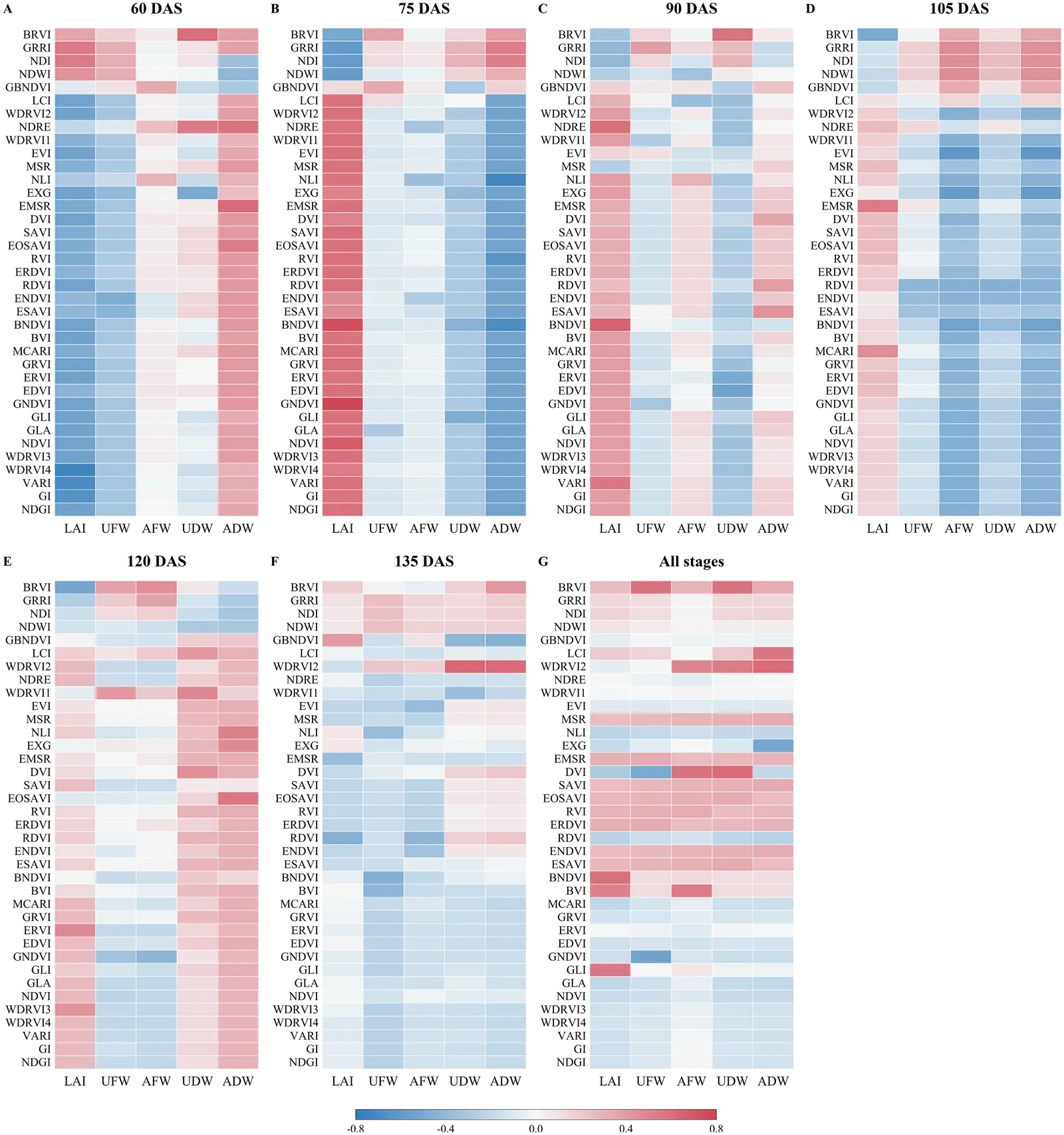
Pearson correlations between VIs and growth traits (LAI, UFW, AFW, UDW, and ADW) at six growth stages and for the pooled whole-season dataset in 2024. **(A–F)** correspond to 60, 75, 90, 105, 120, and 135 DAS, respectively; **(G)** shows correlations calculated after pooling observations from all six DAS.

Pearson correlations between the 37 VIs and five growth traits varied across growth stages (**Figure 4**). Coefficients ranged from -0.74 to 0.78, and the leading VI differed by trait and sampling date. LAI had its strongest VI associations during the early and middle stages. At 75 DAS, GNDVI correlated most closely with LAI (r = 0.78), followed by BNDVI (r = 0.74) and NDVI (r = 0.68).

Fresh biomass correlations were weaker and more variable. UFW was most closely associated with WDRVI1 (r = 0.42), BRVI (r = 0.38), and GNDVI (r = -0.34) at 120 DAS. AFW correlations were concentrated at 105 DAS, led by EVI (r = -0.62), EXG (r = -0.58), and BNDVI (r = -0.54). Dry biomass showed stronger stage dependence. UDW was associated with BRVI at 60 DAS (r = 0.60) and 90 DAS (r = 0.58), WDRVI1 at 120 DAS (r = 0.50), and WDRVI2 at 135 DAS (r = 0.63). For ADW, high absolute correlations occurred at 75 DAS with NLI (r = -0.72), BNDVI (r = -0.68), and RVI (r = -0.64), and at 135 DAS with WDRVI2 (r = 0.62). After pooling observations from all six dates, the whole-season VI-trait correlations ranged from -0.54 to 0.62, indicating clear trait-specific differences in spectral response. For LAI, BNDVI, GLI, and BVI showed the strongest correlations (r = 0.60, 0.56, and 0.52, respectively). For fresh biomass traits, UFW was mainly associated with BRVI, GNDVI, and DVI (r = 0.58, -0.54, and -0.50, respectively), whereas AFW was most closely related to DVI, BVI, and WDRVI2 (r = 0.58, 0.54, and 0.50, respectively). For dry biomass traits, DVI, BRVI, and WDRVI2 showed the strongest associations with UDW (r = 0.62, 0.58, and 0.54, respectively), while WDRVI2, LCI, and EXG were most strongly associated with ADW (r = 0.62, 0.56, and -0.52, respectively).

### 3.3 Stage-specific retained models developed from correlation-screened single-VI inputs

**Figure 5:**
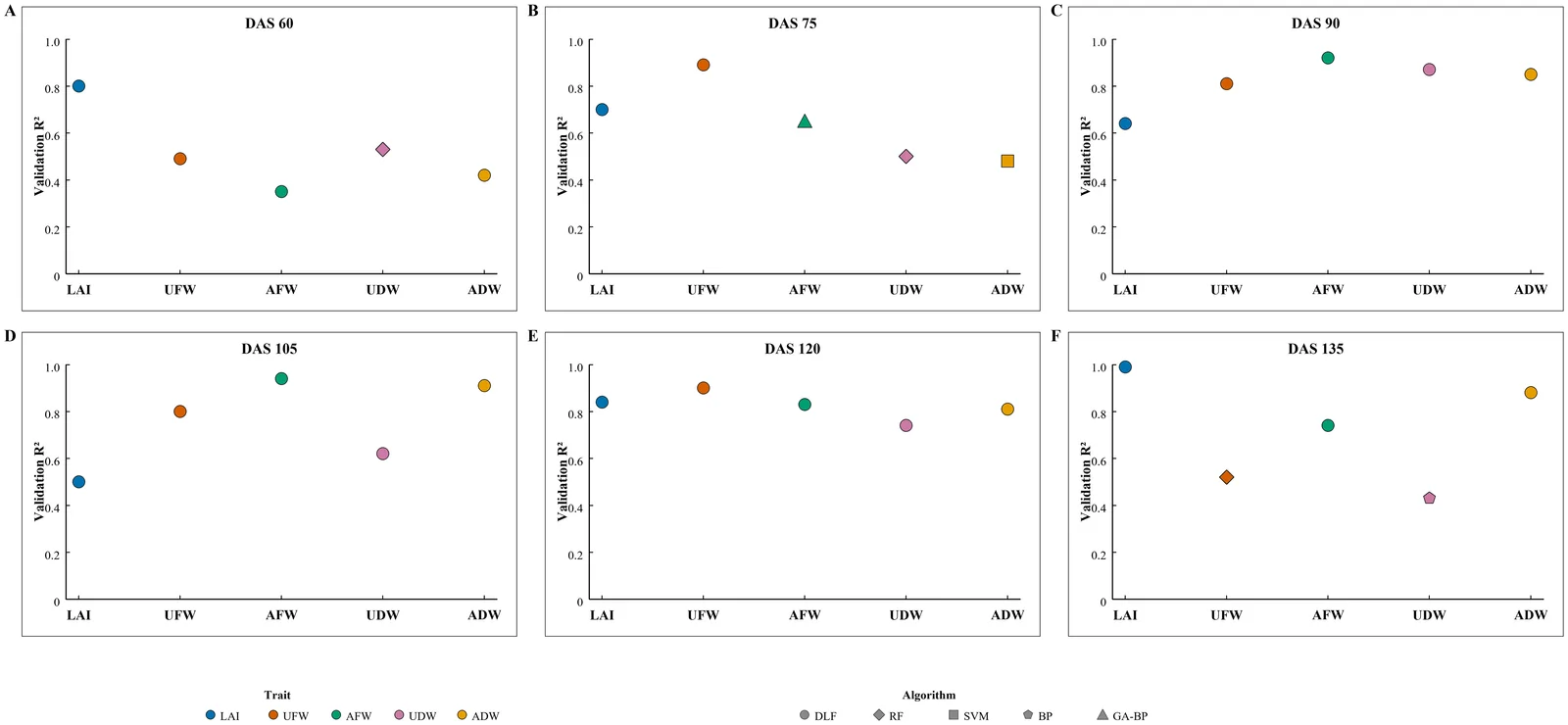
Validation R^2^ distribution of optimal stage-specific single-VI models based on repeated random split validation. **(A-F)** Models corresponding to 60, 75, 90, 105, 120, and 135 DAS. Each dot represents an optimal VI-algorithm combination; dot color indicates growth trait, and dot shape indicates modeling algorithm.

Models built from correlation-screened VI inputs revealed distinct trait- and stage-specific patterns (**Table 2**; **Figure 5**). Across the six DAS, both the retained VI input and the best-performing algorithm changed with target trait. For LAI, DLF was retained at all six stages, but the selected VI changed from VARI at 60 DAS to GNDVI, BNDVI, OSAVI, BRVI, and OSAVI at later stages. Validation performance was lowest at 60 DAS and increased at later stages, reaching its strongest internal validation at 135 DAS with OSAVI input.

**Table 2:** Validation results of retained stage-specific single-VI models.

DAS	Trait	Representative VI	Algorithm	Observed-predicted regression equation	R²	RMSE	MAE	MBE
60 DAS	LAI	VARI	DLF	y=0.3231x+0.9120	0.80	0.01	0.01	0.01
60 DAS	UFW	EMSR	DLF	y=1.2900x+0.0486	0.49	0.10	0.09	-0.02
60 DAS	AFW	EVI	DLF	y=0.2916x+0.8490	0.35	0.42	0.38	-0.08
60 DAS	UDW	BRVI	RF	y=0.3021x+0.5019	0.53	0.11	0.09	0.02
60 DAS	ADW	OSAVI	DLF	y=0.4311x+0.4712	0.42	0.13	0.10	-0.01
75 DAS	LAI	GNDVI	DLF	y=0.3922x+0.8469	0.70	0.02	0.02	0.01
75 DAS	UFW	BRVI	DLF	y=0.5108x+0.6879	0.89	0.36	0.32	0.19
75 DAS	AFW	DVI	GA-BP	y=0.602x+2.2460	0.65	1.10	0.80	-0.18
75 DAS	UDW	GLI	RF	y=0.5498x+0.8903	0.50	0.37	0.29	0.10
75 DAS	ADW	BNDVI	SVM	y=0.458x+0.7738	0.48	0.04	0.03	0.03
90 DAS	LAI	BNDVI	DLF	y=0.2516x+1.144	0.64	0.02	0.02	0.01
90 DAS	UFW	GRRI	DLF	y=0.3755x+1.5910	0.81	0.29	0.19	0.08
90 DAS	AFW	LCI	DLF	y=1.0080x+0.4218	0.92	0.34	0.24	-0.12
90 DAS	UDW	BRVI	DLF	y=0.6819x+0.7390	0.87	0.97	0.75	0.32
90 DAS	ADW	ESAVI	DLF	y=0.3419x+1.6890	0.85	0.38	0.29	0.17
105 DAS	LAI	OSAVI	DLF	y=0.5128x+0.5819	0.50	0.05	0.04	0.03
105 DAS	UFW	RDVI	DLF	y=0.5869x+0.8729	0.80	0.31	0.17	-0.06
105 DAS	AFW	GBNDVI	DLF	y=0.7334x+0.6189	0.94	0.21	0.11	0.05
105 DAS	UDW	RDVI	DLF	y=0.6180x+1.1100	0.62	0.48	0.36	0.16
105 DAS	ADW	GBNDVI	DLF	y=0.7684x+0.3789	0.91	0.28	0.18	0.10
120 DAS	LAI	BRVI	DLF	y=0.3109x+0.8651	0.84	0.02	0.02	0.02
120 DAS	UFW	WDRVI1	DLF	y=0.3860x+1.2900	0.90	0.89	0.58	0.22
120 DAS	AFW	BRVI	DLF	y=0.4130x+1.0980	0.83	1.12	0.74	-0.27
120 DAS	UDW	WDRVI1	DLF	y=0.4585x+0.9018	0.74	0.61	0.33	0.15
120 DAS	ADW	OSAVI	DLF	y=0.3280x+1.4970	0.81	0.01	0.01	0.01
135 DAS	LAI	OSAVI	DLF	y=0.7642x+0.3142	0.99	0.17	0.08	-0.03
135 DAS	UFW	BVI	RF	y=0.6545x+1.1850	0.52	1.35	1.09	-0.36
135 DAS	AFW	RDVI	DLF	y=0.2987x+2.161	0.74	1.06	0.85	0.85
135 DAS	UDW	WDRVI2	BP	y=0.5732x+1.6330	0.43	0.96	0.79	0.57
135 DAS	ADW	WDRVI2	DLF	y=0.8394x+0.6303	0.88	0.48	0.37	0.18

For each DAS-trait combination, the three VIs with the highest absolute correlations were screened as candidate inputs; the retained result reports the best-performing single-VI-algorithm combination. Representative VI denotes the retained VI. Metrics were calculated from validation data.

For fresh biomass, retained models performed best during the middle and late stages. UFW reached its highest validation accuracy at 120 DAS with WDRVI1 input and DLF (R^2^ = 0.90). AFW reached its highest accuracy at 105 DAS with GBNDVI input and DLF (R^2^ = 0.94), while its lowest R^2^ occurred at 60 DAS. For dry biomass, UDW reached its highest validation R^2^ at 90 DAS with BRVI input and DLF (R^2^ = 0.87). ADW performed best at 105 DAS with GBNDVI input and DLF (R^2^ = 0.91) and remained high at 135 DAS with WDRVI2 input and DLF (R^2^ = 0.88). Among the 30 stage-specific trait-stage combinations, DLF was retained most frequently, whereas RF, GA-BP, BP, and SVM were selected in specific cases. For biomass traits, the lowest or near-lowest R^2^ values occurred at 60 DAS: 0.49 for UFW, 0.35 for AFW, 0.53 for UDW, and 0.42 for ADW. Model performance improved during 90–120 DAS. At 135 DAS, LAI and ADW maintained relatively high internal validation performance, whereas UFW and UDW declined to R^2^ = 0.52 and 0.43, respectively.

### 3.4 Validation of representative stage-specific models

**Figure 6:**
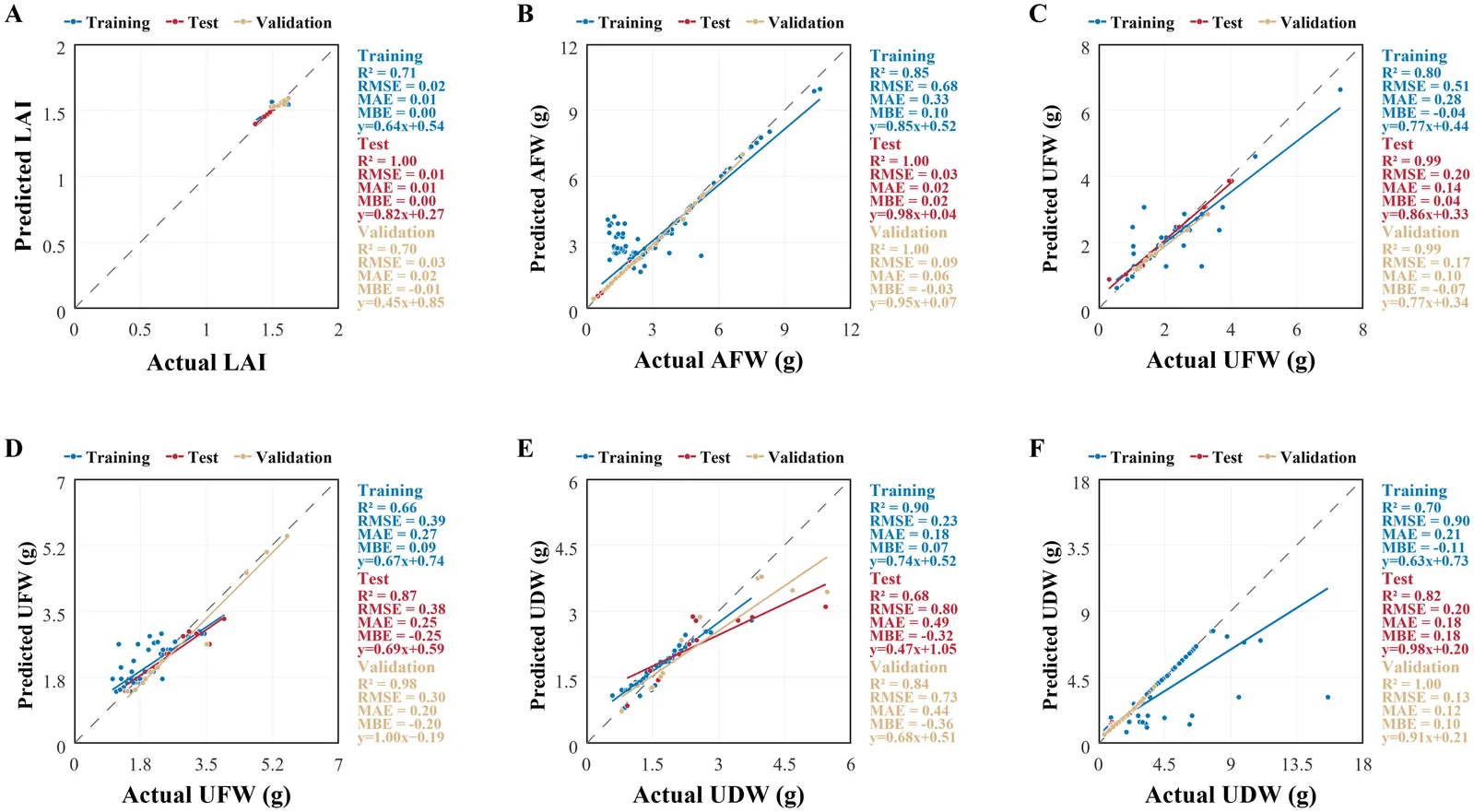
Observed and predicted values for representative stage-specific retained models. **(A–F)** correspond to LAI at 60 DAS with VARI input and DLF modeling, AFW at 75 DAS with DVI input and GA-BP modeling, UFW at 90 DAS with GRRI input and DLF modeling, UFW at 105 DAS with RDVI input and DLF modeling, UDW at 120 DAS with WDRVI1 input and DLF modeling, and UDW at 135 DAS with WDRVI2 input and BP modeling, respectively. Blue, red, and beige symbols indicate training, test, and validation subsets; dashed lines indicate 1:1 relationships.

Observed-predicted relationships for the six representative retained models illustrated the stage-specific results without repeating all 30 model combinations (**Figure 6**). The selected cases covered LAI at 60 DAS, AFW at 75 DAS, UFW at 90 and 105 DAS, and UDW at 120 and 135 DAS. The early-stage LAI model fitted better than the early-stage AFW model. LAI at 60 DAS was estimated with VARI input and DLF (R^2^ = 0.80; RMSE = 0.01), whereas AFW at 75 DAS was estimated with DVI input and GA-BP (R^2^ = 0.65; RMSE = 1.10).

The two UFW models selected at 90 and 105 DAS showed comparable validation accuracy. UFW with GRRI input and DLF at 90 DAS yielded R^2^ = 0.81, RMSE = 0.29, MAE = 0.19, and MBE = 0.08. UFW with RDVI input and DLF at 105 DAS yielded R^2^ = 0.80, RMSE = 0.31, MAE = 0.17, and MBE = -0.06. UDW accuracy differed between 120 and 135 DAS. UDW with WDRVI1 input and DLF at 120 DAS reached R^2^ = 0.74 and RMSE = 0.61, whereas UDW with WDRVI2 input and BP at 135 DAS reached R^2^ = 0.43 and RMSE = 0.96. The 135 DAS UDW model had the lowest R^2^ among the representative cases.

### 3.5 Whole-season model performance and pooled internal validation

**Figure 7:**
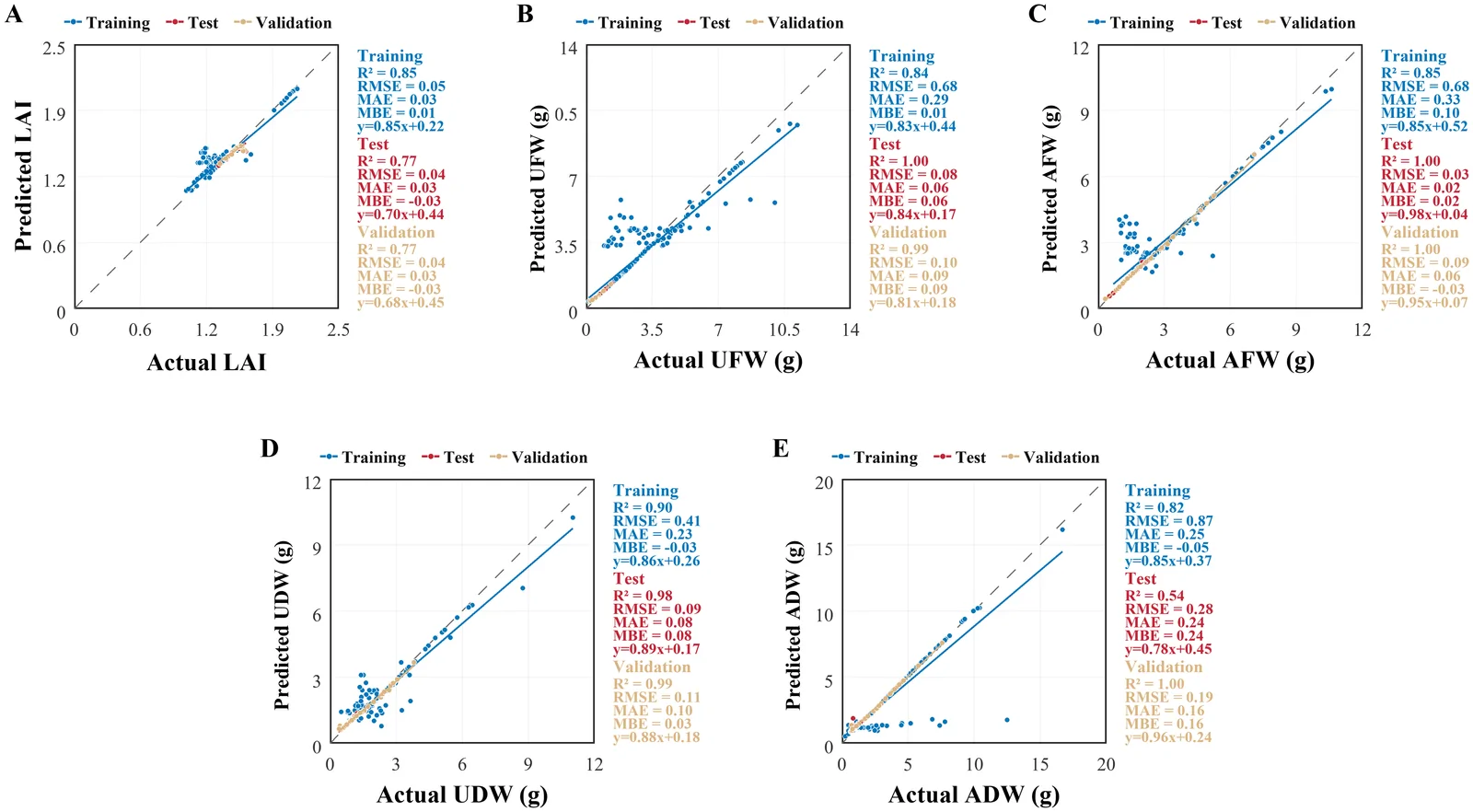
Observed and predicted values for retained whole-season models of *A. membranaceus* growth traits. **(A–E)** correspond to LAI with BNDVI input and BP modeling, UFW with BRVI input and DLF modeling, AFW with DVI input and DLF modeling, UDW with DVI input and DLF modeling, and ADW with WDRVI2 input and DLF modeling, respectively.

Whole-season models developed from the pooled dataset revealed trait-specific performance patterns (**Table 3**; **Figure 7**). Retained VI inputs and algorithms differed among the five target traits and were consistent with the dominant whole-season associations summarized in **Figure 4G**. LAI and UDW had the highest whole-season accuracies. LAI with BNDVI input and BP reached R^2^ = 0.96, RMSE = 0.01, MAE = 0.003, and MBE = -0.003. UDW with DVI input and DLF reached R^2^ = 0.93, RMSE = 0.39, MAE = 0.19, and MBE = 0.12.

**Table 3:** Validation results of retained whole-season single-VI models.

Trait	Representative VI	Algorithm	Observed-predicted regression equation	R²	RMSE	MAE	MBE
LAI	BNDVI	BP	y=1.003x-0.0034	0.96	0.01	0.003	-0.003
LAI	GLI	DLF	y=0.924x+0.0201	0.91	0.03	0.02	0.01
LAI	BVI	DLF	y=0.8414x+1.2010	0.88	0.04	0.02	0.01
UFW	BRVI	DLF	y=0.8488x+0.4464	0.85	0.55	0.23	0.16
UFW	GNDVI	SVM	y=0.7787x+0.0792	0.81	0.03	0.01	-0.01
UFW	DVI	SVM	y=0.8625x-4.2560	0.73	2.29	1.18	0.01
AFW	DVI	DLF	y=0.7357x-8.0880	0.87	0.55	0.24	0.12
AFW	BVI	DLF	y=0.7727x+1.7990	0.87	0.70	0.11	0.05
AFW	WDRVI2	GA-BP	y=0.7316x-0.1314	0.86	0.08	0.05	0.02
UDW	DVI	DLF	y=0.8900x-2.6580	0.93	0.39	0.19	0.12
UDW	BRVI	DLF	y=0.7902x+0.4308	0.89	0.72	0.27	0.08
UDW	WDRVI2	GA-BP	y=0.9473x-0.0246	0.89	0.04	0.02	0.02
ADW	WDRVI2	DLF	y=0.7583x-0.1274	0.88	0.41	0.23	0.17
ADW	LCI	SVM	y=0.7994x+0.029	0.85	0.01	0.01	-0.01
ADW	EXG	GA-BP	y=0.7873x+3.9150	0.82	1.66	0.87	0.03

For each agronomic trait, the three VIs with the highest whole-season pooled correlations were adopted as candidate inputs. All retained models are sorted by validation R^2^, and the representative VI corresponds to the single vegetation index adopted as model input.

AFW and ADW showed intermediate performance in the pooled dataset. AFW with DVI input and DLF and AFW with BVI input and DLF both reached R^2^ = 0.87, while AFW with WDRVI2 input and GA-BP reached R^2^ = 0.86. ADW with WDRVI2 input and DLF achieved the highest R^2^ (0.88), followed by LCI input with DLF modeling (R^2^ = 0.85) and EXG input with GA-BP modeling (R^2^ = 0.82). UFW had the lowest whole-season R^2^ among the five traits. UFW with BRVI input and DLF performed best (R^2^ = 0.85; RMSE = 0.55; MAE = 0.23; MBE = 0.16), followed by UFW models using GNDVI input with SVM modeling (R^2^ = 0.81) and DVI input with SVM modeling (R^2^ = 0.73). For the best whole-season models, the observed-predicted panels corresponded to LAI with BNDVI input and BP modeling, UFW with BRVI input and DLF modeling, AFW and UDW with DVI input and DLF modeling, and ADW with WDRVI2 input and DLF modeling (**Figure 7**). LAI and UDW points were concentrated near the 1:1 line. AFW and ADW showed more stable observed-predicted distributions, whereas UFW was more dispersed. Compared with UDW, the greater dispersion of UFW around the 1:1 line likely reflected variation in root water content at harvest, which introduced additional variability into fresh-weight measurements.

## 4 Discussion

### 4.1 Effects of irrigation-nitrogen treatments on LAI and biomass accumulation

Water and nitrogen jointly regulate LAI development, photosynthetic carbon supply, nitrogen assimilation, and biomass allocation. Under adequate water supply, additional nitrogen can promote leaf expansion and photosynthetic capacity, whereas water limitation restricts stomatal conductance, nutrient transport, and nitrogen assimilation. Conversely, greater irrigation under limited nitrogen may increase tissue hydration without supporting proportional dry-matter formation. These coupled constraints help explain why treatment separation became more evident after 90 DAS rather than following a simple input-response gradient. Photoassimilate export from leaves and allocation among shoots and roots are regulated source-sink processes that change with resource availability (Ainsworth and Bush, 2011; Poorter et al., 2012), and nitrogen-driven changes in plant height, root morphology, and biomass have been reported in *A. membranaceus* (Wang et al., 2020).

Against this physiological background, W1N5 produced the highest final LAI, whereas W2N2 produced the greatest above- and belowground biomass. Abundant water and nitrogen under W1N5 likely favored the expansion and maintenance of photosynthetic leaf area; however, the larger LAI did not translate proportionally into root dry-matter accumulation. This interpretation is consistent with cassava evidence showing that excessive nitrogen can increase aboveground resource demand while compromising carbohydrate allocation to storage roots (Omondi et al., 2019). Nevertheless, carbon translocation was not measured directly, so this source-sink interpretation remains mechanistic rather than causal. W2N2 (22.5 mm per irrigation event and 150 kg N ha⁻¹) more closely balanced source development with sink demand and reached the maximum UFW, AFW, UDW, and ADW at 135 DAS, whereas W2N4 produced the lowest final biomass. Studies in *P. notoginseng* and *G. uralensis* similarly show that coordinated water and fertilizer supply can improve root yield and dry-matter deposition, whereas imbalanced inputs may reduce growth efficiency (Li et al., 2021; Tuo et al., 2025; Abudurezike et al., 2024). Thus, W2N2 should be interpreted as the biomass-optimal treatment under this single field season rather than as a universally optimal prescription.

The contrasting treatment responses also demonstrate that irrigation and nitrogen effects cannot be evaluated independently. Increasing nitrogen under low water availability is unlikely to overcome restrictions on leaf gas exchange and nutrient transport, while increasing irrigation without adequate nitrogen does not ensure equivalent biomass formation. Optimized water-nitrogen management in maize has likewise improved root development, nutrient uptake, dry-matter accumulation, and yield relative to a higher-nitrogen treatment under the same high soil-moisture regime (Chi et al., 2025). Agronomic management should therefore coordinate both inputs and prioritize the harvested root response rather than LAI alone. Because this experiment covered only the first growing season of a perennial medicinal crop, the identified biomass optimum requires confirmation over subsequent growth years.

### 4.2 Growth-stage variation in VI sensitivity to LAI and biomass traits

Early-stage spectral sensitivity was governed by low LAI and residual soil exposure. At 75 DAS, GNDVI correlated most closely with LAI (r = 0.78), followed by BNDVI and NDVI, whereas biomass correlations remained weaker. Green and near-infrared combinations respond to chlorophyll-related absorption and leaf-area development, but mixed soil-plant pixels reduce their ability to distinguish small biomass differences (Berni et al., 2009; Chatterjee et al., 2025). Soil-adjusted indices are designed to reduce soil-brightness effects (Rondeaux et al., 1996). Among the indices evaluated here, however, only OSAVI was retained at 60 DAS, for ADW, and its validation R^2^ was 0.42; SAVI was not retained, and MSAVI was not included in the 37-index set. Soil adjustment therefore did not fully compensate for the combined effects of low LAI, limited biomass contrast, and soil-vegetation mixing at 60 DAS. Early-stage UAV studies likewise show that spectral similarity between seedlings and bare soil can constrain crop extraction even when multispectral VIs and machine learning are combined (Li et al., 2024).

As LAI increased, the dominant spectral information shifted toward biomass accumulation and dry-matter deposition. Red reflectance is influenced by pigment absorption, red-edge reflectance responds to changes in chlorophyll status and leaf area, and near-infrared reflectance is shaped by multiple scattering within leaves and the plant stand. Indices combining these regions can therefore respond to changes in photosynthetic tissue, structure, and water status that accompany biomass formation. BRVI was sensitive to UDW at 60 and 90 DAS and to UFW at 75 and 120 DAS, whereas WDRVI2 was strongly related to both UDW and ADW at 135 DAS. These changes are consistent with a progression from early leaf-area establishment to stronger dry-matter accumulation, but they do not imply that aboveground spectra directly measured roots; the associations remained indirect expressions of coordinated whole-plant growth.

No single VI remained optimal across all traits and stages, which has direct implications for field deployment. GNDVI and related greenness indices were most informative for early LAI assessment; BRVI contributed more strongly to selected fresh- and dry-biomass traits from 60 to 120 DAS; and WDRVI2 was more informative for late dry-biomass estimation. Comparable changes in UAV feature contribution have been reported in phenology-aware LAI, yield, and biomass studies, including Qiao et al. (2022) and Prey et al. (2022), while biomass-focused work also emphasizes stage-aware feature screening (Peng et al., 2024; Dlamini et al., 2025).Multi-period rapeseed mapping similarly showed that the importance of optical and phenotypic metrics changed among seedling, bolting, and early flowering stages (Sun et al., 2024). Operational monitoring should therefore match the VI and model to the target trait and observation date. Stable radiometric correction, consistent flight geometry, soil and shadow masking, and local calibration remain necessary because soil brightness, plant density, tissue water status, and site conditions can alter VI sensitivity.

### 4.3 Stage-specific modeling performance across growth stages

The stage-specific models demonstrate that algorithm suitability depends jointly on the target trait and observation date (**Table 2**; **Figure 5**). At 60 DAS, validation R^2^ values for UFW, AFW, UDW, and ADW were 0.49, 0.35, 0.53, and 0.42, respectively. Low LAI, residual soil exposure, and limited biomass contrast constrained the information available to all algorithms; the modest OSAVI result discussed above shows that soil adjustment alone did not remove this limitation. Performance generally improved during 90–120 DAS as treatment-related trait gradients widened. At 135 DAS, LAI and ADW remained comparatively well estimated, whereas UFW and UDW declined, confirming that model behavior differed among traits rather than following a uniform seasonal trajectory.

The representative retained partitions in **Figure 6** illustrate this trait-stage variation without repeating all 30 combinations summarized in **Table 2**. DLF was retained for the illustrated LAI, UFW, and 120 DAS UDW cases, whereas GA-BP and BP were retained for AFW at 75 DAS and UDW at 135 DAS, respectively. The changing VI inputs and algorithms indicate that the statistical relation available to each model evolved as leaf-area variation, tissue water status, and dry-matter accumulation contributed differently to the multispectral signal.

DLF was retained in 24 of the 30 stage-specific models, giving it the highest selection frequency under the present dataset and validation strategy. Integrating heterogeneous learners can reduce dependence on a single functional form and improve fitting when spectral-trait relationships are nonlinear. Nevertheless, RF, GA-BP, BP, and SVM remained preferable in specific trait-stage combinations, so DLF should be regarded as the most consistently selected framework rather than a universally superior algorithm. Comparable changes in algorithm ranking occur in UAV-based LAI and biomass studies using RF, SVM, neural networks, and ensemble methods (Yuan et al., 2017; Cheng et al., 2024), and recent phenotyping studies also report changes in performance with growth stage and feature type (Chatterjee et al., 2025; Li et al., 2025c). The high validation values reported here should therefore be interpreted as internal performance under repeated random partitioning rather than independent evidence of transferability.

### 4.4 Whole-season modeling performance based on combined-stage data

Whole-season models based on pooled observations provide a seasonal view of monitoring capacity (**Table 3**; **Figure 7**). Combining dates broadened the observed ranges of LAI and biomass, and the retained models captured these broad seasonal trajectories. BNDVI with BP was retained for LAI, whereas DLF paired with BRVI, DVI, and WDRVI2 was retained for UFW, AFW/UDW, and ADW, respectively. UDW showed the strongest whole-season performance (R^2^ = 0.93; RMSE = 0.39). Pooled and multi-temporal UAV studies likewise show that wider phenological and trait gradients can improve biomass or yield prediction when spectral responses are nonlinear (Qiao et al., 2022; Chatterjee et al., 2025). Related multi-date studies also demonstrate the value of combining temporal and multisource information for crop biomass and yield estimation (Amorim et al., 2022; Maimaitijiang et al., 2020; Shan et al., 2026).

The contrast between stage-specific and whole-season results also defines an important limitation. High accuracy in the pooled dataset partly reflects separation among phenological stages and does not guarantee equally strong discrimination within each date (Prey et al., 2022; Chatterjee et al., 2025). In addition, the experiment covered one growing season, one site, and internal random partitions; independent data from other years, cultivars, soil conditions, and pedoclimatic zones were not available. Accordingly, fixed seasonal equations should not be transferred directly to other production environments. Comparable specialty-crop studies show that combining UAV observations with soil or multisource spatial data can improve the operational value of machine-learning predictions (Demir et al., 2024; González-Vivar et al., 2026). After external validation, the stage-aware VI-model rules could be incorporated into farm-management software to schedule UAV surveys and provide plot-level LAI and biomass estimates at the most informative observation windows. Multi-year and multi-location trials should test both the stage-specific and pooled models before operational deployment, consistent with the need for robust validation of nonlinear ensemble frameworks (Li et al., 2022b; Cheng et al., 2024).

At broader spatial scales, multi-crop modeling that integrates high-resolution agro-climatic simulations also reveals crop-specific prediction errors and substantial uncertainty in future yield projections (Kaya et al., 2026). These results further indicate that model transfer requires environmental covariates and independent evaluation beyond the calibration domain.

## 5 Conclusions

UAV multispectral sensing provided a feasible approach for monitoring leaf area index (LAI) and biomass dynamics of *A. membranaceus* under 15 irrigation-nitrogen treatments across six growth stages. The treatment optima differed between LAI and biomass: W1N5 maximized LAI, whereas W2N2 maximized both aboveground and underground biomass. This contrast demonstrates that greater leaf expansion under the highest water and nitrogen inputs does not necessarily result in greater biomass production. Under the conditions of this experiment, W2N2 achieved a more favorable balance between LAI development and biomass accumulation, highlighting the importance of coordinating irrigation and nitrogen supply rather than maximizing either input.

The relationships between vegetation indices (VIs) and growth traits varied across growth stages. Biomass estimation was comparatively weak during early establishment but improved as growth and treatment differences became more pronounced. Consequently, no single VI-algorithm combination was consistently optimal for all traits and stages, supporting the use of trait- and stage-adaptive VI selection instead of a fixed input throughout the growing season.

Decision-level fusion exhibited the most consistent overall performance by integrating complementary information from the individual algorithms. The favorable whole-season estimation of underground dry weight further indicates that aboveground multispectral observations can provide an indirect measure of seasonal root dry-matter accumulation.

Collectively, these findings establish a stage-adaptive framework for UAV-based monitoring of *A. membranaceus* and provide a technical basis for precision irrigation and nitrogen management. Following independent external validation, the retained VI-model combinations could be incorporated into farm decision-support systems to provide stage-specific estimates of LAI and biomass and support timely management decisions. Future studies should evaluate model stability and transferability through independent multi-year and multi-location field trials before operational deployment.

Because the present models were developed from a single growing season at one experimental site, their performance should currently be interpreted as site- and season-specific. Future research should evaluate model stability and transferability using independent multi-year and multi-location datasets encompassing contrasting soil and climatic conditions before operational deployment.

## Data Availability

The original contributions presented in the study are included in the article/supplementary material. Further inquiries can be directed to the corresponding authors.
